# Possible Role of Platelet GluR1 Receptors in Comorbid Depression and Cardiovascular Disease

**DOI:** 10.1155/2009/424728

**Published:** 2009-08-16

**Authors:** Hu Chen

**Affiliations:** Department of Psychiatry, Psychiatric Institute, University of Illinois at Chicago, 1601 West Taylor Street, MC912, Chicago, IL 60612, USA

## Abstract

The exact nature of the comorbidity between cardiovascular disease (CVD) and major depressive disorder (MDD) is poorly understood. The proposed mechanisms include various biochemical and molecular pathways as well as health behaviors such as physical inactivity. One possible link between MDD and CVD is increased platelet activity and blood viscosity. Recently, it was discovered that platelets express functional subtype of alpha-amino-3-hydroxy-5-methyl-4-isoxazolepropionic acid (AMPA) receptors, for example, glutamate receptor 1 (GluR1). Here, I propose that this type of AMPA receptor could play a role in comorbid MDD and CVD, and antidepressants may interfere with platelet activation via direct or indirect effects on platelet GluR1 phosphorylation. Testing this hypothesis could provide a novel view on the pathobiological mechanisms of comorbid MDD and CVD. With respect to the recently discovered role of AMPA receptors in regulating platelet activation and thrombosis, it appears that the information about the putative effects of psychoactive AMPA-modifying drugs on platelet AMPA receptors would be critical in evaluating the putative effects of such drugs on CVD.

## 1. Introduction

Epidemiological studies have identified a high incidence of comorbidity between cardiovascular disease (CVD) and major depressive disorder (MDD). These studies note that patients with MDD are at greater risk of developing CVD [1]. Further, they prompted the American Heart Association to recommend routine screening for depression in patients with coronary heart disease, a recommendation that still needs to be fully implemented [2]. The exact nature of the MDD-CVD association is poorly understood. The proposed mechanisms of this link include various biological, that is, biochemical and molecular pathways as well as the hypothesis that the association between depressive symptoms and cardiovascular events could be driven by health behaviors, especially physical inactivity [3].

One prominently hypothesized link between MDD and CVD includes increased platelet activity and blood viscosity in these patients [4–7]. It has been suggested that serotonin and its molecular/cellular targets are altered in patients with MDD in a way that leads to increased platelet activation and enhanced risk for CVD [4]. Several studies have shown that this abnormal activation of platelets can be attenuated by psychotherapy and by treatment with selective serotonin reuptake inhibitors (SSRIs) [8–10].

Recently, it was discovered that platelets express the alpha-amino-3-hydroxy-5-methyl-4-isoxazolepropionic acid (AMPA) receptors for the excitatory neurotransmitter glutamate [11]. Furthermore, these authors showed that an AMPA receptor subtype, glutamate receptor 1 (GluR1), mediates the action of glutamate as a regulator of platelet activation, and they suggested that the GluR1 receptor is a novel antithrombotic target. Here, I propose that this type of glutamate receptor could play a role in comorbid MDD and CVD.

## 2. Presentation of the Hypothesis

Ionotropic glutamate receptors are ligand-gated ion channels that can be subdivided into three classes: NMDA (*N*-methyl-d-aspartate), kainate, and AMPA receptors. GluR1, one of AMPA receptor subunits, mediates transmission and plasticity at excitatory synapses in a manner which is positively regulated by phosphorylation at Ser845-GluR1, a protein kinase A (PKA) site, and at Ser831-GluR1, a calcium/calmodulin-dependent kinase II (CaMKII) or protein kinase C (PKC) site [12, 13].

Recent work by Morrell et al. [11] demonstrated that activated platelets release glutamate and express GluR1 AMPA subunits; glutamate increases agonist-induced platelet activation. Furthermore, glutamate binding to the AMPA receptors depolarized platelets (an important step in platelet activation), and platelets treated with an AMPAR antagonist or platelets derived from GluR1 knockout mice were resistant to the effects of AMPA. In addition, mice lacking GluR1 have a prolonged time to thrombosis in vivo [11]. Thus, activation of GluR1 plays a role in accelerating thrombus formation and may contribute to development of CVD.

It has been noted that plasma concentrations of glutamate are altered in MDD. Plasma levels of glutamate increased with the severity of depression [14], and antidepressant therapy was capable of reducing these levels [15]. On the other hand, measurements of the platelet response to glutamate revealed that platelet glutamate receptors are supersensitive in MDD [16].

Considering the crucial role of increased GluR1 phosphorylation in the process of membrane insertion of these subunits and in the consequent increased activity of these AMPA receptors [17], and also considering known alterations of protein kinase systems (including PKA) in mood disorders [18], I hypothesize that altered platelet GluR1 phosphorylation in MDD may contribute to comorbid MDD and CVD.

## 3. Antidepressants and GluR1 Phosphorylation

The trafficking of the GluR1 from intracellular pools to cell membranes is guided by a well-regulated pattern of receptor phosphorylation. It has been suggested that phosphorylation of GluR1 is involved in antidepressant-like actions [19]. However, the data on medication-induced alterations of GluR1 phosphorylation are complex. Both an increase and a decrease of GluR1 phosphorylation were observed in response to antidepressant treatment, and various antidepressants influence the phosphorylation of different sites of GluR1. For example, Svenningsson et al. [20] reported that treatment with fluoxetine increases phosphorylation at the Ser845 but not the Ser831 site. In addition, another antidepressant, tianeptine, which enhances the reuptake of serotonin instead of inhibiting it, increased Ser831 phosphorylation in the frontal cortex and CA3 region of the hippocampus but increased Ser845 phosphorylation only in the CA3 region. Behavioral analyses showed that mice bearing point mutations at both Ser831 and Ser845 when treated with saline exhibit increased immobility in the tail suspension test (i.e., depression-like behavior) compared to their wild-type counterparts. Chronic tianeptine treatment reduced this immobility in wild-type mice but not in phosphomutant GluR1 mice [21]. Recent findings in animal and human studies suggest that minocycline has antidepressant-like neuroprotective effects, and it has been shown to act as an antidepressant in a rat model of depression [22]. In vitro and in vivo studies showed that minocycline induces GluR1 phosphorylation at both Ser845 and Ser831, and it increases the surface content of GluR1 [23]. In contrast, a subanesthetic dose of ketamine, which causes acute and sustained antidepressant-like effects, significantly lowered the levels of phosphorylated GluR1 at Ser845 [24].

## 4. Platelet Activation and Antidepressants

Antidepressants, particularly SSRIs, have been associated with abnormal bleeding. Drugs with the highest degree of serotonin reuptake inhibition, for example, fluoxetine, paroxetine, and sertraline, are more frequently associated with abnormal bleeding and modifications of hemostasis markers [25]. Thus, it has been inferred that SSRIs may also bestow protection from myocardial infarction, even compared to other classes of antidepressants. This has been demonstrated in some clinical studies [26]. More recent studies suggest that SSRIs exert a complex sequence of effects relevant to platelet activation and that, in some instances, they may even be prothrombotic [27]. Since the known antidepressants, in addition to their main effects (e.g., serotonin reuptake inhibition), also affect multiple molecular pathways (e.g., GluR1 signaling and phosphorylation), I hypothesize that these drugs may interfere with platelet activation via direct or indirect effects on platelet GluR1 phosphorylation.

## 5. Testing the Hypothesis

The hypothesis that altered platelet GluR1 phosphorylation in MDD may contribute to comorbid MDD, and CVD would be best tested in a clinical setting. Since several studies have already established alterations of glutamate levels and receptor activation in MDD patients versus controls, this approach could be used to assess the phosphorylation of platelet GluR1. Moreover, future clinical studies could focus on patients with comorbid MDD and CVD. In addition, in vitro studies with human platelets are warranted to verify whether alterations of platelet GluR1 phosphorylation (e.g., induced by kinase inhibitors and activators) influence the extent of GluR1 insertion into the platelet membrane and whether they influence platelet activation.

The hypothesis that drugs used for treatment of MDD may interfere with platelet activation could be tested both in animal models and in a clinical setting. It would be useful to compare the effects of SSRIs with novel antidepressants developed specifically to target AMPA receptors. These studies would be primarily exploratory because the predicted mechanisms of action of these drugs on GluR1 receptors could significantly differ in the central nervous system (CNS) and in blood cells such as platelets.

With respect to the recently discovered role of AMPA receptors in regulating platelet activation and possibly playing a significant role in thrombosis, it appears that the information about the putative effects of CNS AMPA-acting drugs on platelet AMPA receptors would be critical in evaluating the putative effects of such drugs on CVD. More generally, testing the here-proposed hypothesis could provide a novel view on the pathobiological mechanisms of comorbid MDD and CVD.
